# Fracture of the Odontoid Process—A Clinical Lecture

**Published:** 1882-12-20

**Authors:** Stephen Smith

**Affiliations:** New York; Professor of Clinical Surgery, in the University of New York


### FRACTURE OF THE ODONTOID PROCESS.—A CLINICAL LECTURE.

BY STEPHEN SMITH, M. D.,

Professor of Clinical Surgery, in the University of New York.

Here is a man whom I show you because he presents an interesting example of a form of fracture usually fatal, but here resulting in recovery. This man broke his neck last year, and that, too, in a very dangerous place, namely: about the location of the first cervical vertebra; and now, if you will put your finger into his throat you will be able to feel the first cervical vertebra projecting into the back part of the mouth. This is probably a case of fracture of the odontoid process, and most cases of this kind have been fatal, until lately, and death was instantaneous, just as it is in an animal whose medulla has been broken up by the operation of pithing, as it is called.

The history of this case is, that last December this man fell from a height upon the deck of an ice barge, and he struck on his neck, and when he was taken up he was found to be partially paralyzed in his arms, and now he is bearing the effects of this paresis in a permanent contraction of the muscles. He was taken to the hospital and he was so paralyzed that he could not sit up in bed, and quiet was insisted upon. He gradually, however, gained more and more use of his limbs, and his head became firmly fastened to his neck with the chin bent downward upon the chest, and so rigidly that he could not move his head from side to side, or up and down.

These cases of fracture of the neck present a very peculiar history. It was once supposed that fracture of the odontoid process was always immediately fatal, and that this was the real cause of death in cases of hanging; but it has recently been proved that the accident may occur and the man still live and go about his business, and yet, finally, die suddenly from some accident, such as being hit upon the head. Thus Dr. Parker tells of a case of a milkman in his city who came from Long Island to sell milk. One day he was thrown out of his wagon upon his head, but he got up and then found that his head was loose and that he could not hold it up nor turn it from side to side, but he steadied it as best he could with his hand, and then got into his wagon and drove home again. For the next three or four days he could not lie down or get up without his head moving about, unless he steadied it with his hands. He then went and saw Dr. Parker, and he, and all who saw the case, were greatly surprised because this accident had always been thought to be fatal. That man finally got so well that he resumed his milk business, and as he drove around he would have to hold his hand upon his head to steady it whenever he drove over a rough place where there was much jolting. So he went on for six months, and then, after a hard day's work, he suddenly fell dead at the table, his head dropping forward upon his chest. The specimen of this fracture is now preserved in a museum.

Recovery takes place in these cases by the formation of an ankylosis between the vertebræ at the seat of the fracture, so you might easily kill this man instantly by striking him upon the head, and so breaking up the adhesions which have formed. In this case we have exactly the same condition that existed in a case in this hospital, which I found when I came on duty here five years ago. I found his head drawn up with his chin projecting, and he was paralyzed from his neck downwards, and he was emaciated almost to a skeleton, and was suffering intensely. He had fallen in some way and struck upon his head, and immediately afterwards he felt this peculiar looseness of the head, and he went home, and after resting for three or four days he resumed his business at the carpenter's trade for a time. Then the paralysis came on, and he went to the hospital, where he stayed for the next six or eight months, and then he died with his head thrown back and his chin out. I found, upon examination, that the atlas had slid forward so that the spinal cord was pressed upon, and this caused his death. But he had a fractured odontoid process, and yet he had continued his work for some time, so it was proved that a man may recover from this accident. At that time I collected a series of thirty-two cases of this nature which had been overlooked in the medical publications, and in some of these no odontoid process could be found, and there were two or three cases among them where the odontoid process was perfectly movable upon the atlas by an articulation with it.

It seems to me perfectly evident that this man is suffering from a fracture of the odontoid process. In treating this fracture we have tried a number of diffierent splints, but we have found none which answers so well as to keep the patient sitting in a chair with a cross piece behind him to which his head is bound so as to steady it. This man was treated so until he gradually became so improved that he could walk around the wards without his head becoming loose, and now he can even run a little.

Observe the evidence of spinal injury presented by his hands. You see there is an unusual thickening of the joints of the fingers, and a loss of action with permanent contraction of some of the muscles, due to the injury of the spinal nerves. You see the man cannot turn his head around at all, and this is diagnostic almost of all these cases. If he should accidentally trip and fall there would probably be a sudden displacement of these bones resulting in immediate death.—*Medical Gazette.*

---

**Pulvis Doveri.**—The Canadian Journal of Medical Science says: People whose "inward griefs and peristaltic woes" have been relieved by the powder of Dover, do not generally know to whom they are indebted for this excellent compound. Doctor Dover was a friend and probably pupil of the great Sydenham. He commenced practice in Bristol, where, having made some money, he longed to make more. The Roll of the College of Physicians tells us that he joined with some merchants in fitting out two privateers for the South Seas, in one of which, the "Duke," he himself sailed from Bristol, 2d August, 1708. On the passage out they touched at the Island of Juan Fernandez, where Dover, on the 2d of February, 1708–9, found Alexander Selkirk, who had been alone on the island for four years and four months, and whom Dover brought away in the "Duke." In the April following Dover took Ginaguil, a city or town of Peru, by storm. In December, 1709, the two privateers took a large and valuable prize, a ship of 20 guns and 190 men, in which Dover removed from the "Duke," taking Alexander Selkirk with him as master, and finally reaching England in October, 1711. After this cruise Dr. Dover removed to London, where his practice soon became great. His patients, and the apothecaries who wished to consult him, addressed their letters to the Jerusalem coffee house, where at certain hours of the day he received most of his patients.—*Mich. Med. News.*

